# Editorial: Multi-omic approaches decipher the pathogenesis of nervous system diseases and identify potential therapeutic drugs

**DOI:** 10.3389/fgene.2024.1520148

**Published:** 2024-12-10

**Authors:** Robert Friedman, Yasin Mamatjan, Cuiping Pan, Patrícia Pelufo Silveira, Margarita Zachariou

**Affiliations:** ^1^ Department of Biological Sciences, University of South Carolina (Retired), Columbia, SC, United States; ^2^ Department of Engineering, Thompson Rivers University, Kamloops, BC, Canada; ^3^ Center for Intelligent Medicine Research, Greater Bay Area Institute of Precision Medicine (Guangzhou), Fudan University, Guangzhou, China; ^4^ Ludmer Centre for Neuroinformatics and Mental Health, Douglas Hospital Research Centre, McGill University, Montreal, QC, Canada; ^5^ Bioinformatics Department, The Cyprus Institute of Neurology and Genetics, Nicosia, Cyprus

**Keywords:** neurological disease, pathogenesis, multiomics, high-throughput sequencing, machine learning

Diseases of the nervous system (both central and peripheral) involve complex underlying molecular mechanisms and have a detrimental impact on the survival and quality of patient life. Multi-omic approaches provide powerful tools for uncovering complex networks of disease pathogenesis and enable the screening of key potential biomarkers and therapeutic drug targets. The term “omics”, proposed 4 decades ago, denotes a discipline within the biological sciences that features the employment of high-throughput technologies to study biomolecules systematically. It includes, but is not limited to, application to the genome, proteome, transcriptome, and metabolome (Vailati-Riboni et al., 2017). These various -omics technologies have revolutionized biomedical research, and in combination, they will no doubt lead to a further understanding of the pathologies of the nervous system. To encourage the scientific community to employ multi-omic approaches in studies of nervous system pathology, we selected several articles for this Research Topic that serve as ideal examples.

This Research Topic includes studies in murine models for mechanical allodynia in type 1 diabetes (Chen et al.), temporal lobe epilepsy (Huang et al.), pathological anxiety (Gigliotta et al.), and retinal myopia (Pan et al.). Furthermore, we included a clinical study of a molecular diagnosis for episodic ataxia (Audet et al.). Altogether, these studies utilized various types of “-omics” data and bioinformatic techniques to predict genetic biomarkers of disease and potential targets for drug treatment, thereby bridging the translational gap between the basic sciences and clinical applicability.

In the first article, Chen et al. investigated in rat models of type 1 diabetes the pathogenic cause of mechanical allodynia, i.e., pain evoked by light touch, a leading clinical symptom of painful diabetic peripheral neuropathy. Their study associated allodynia with a disorder of lipid metabolism, resulting in lipid accumulation and myelin sheath degeneration. In particular, correlations in the lipidome and transcriptome led to the identification of the downregulation of the gene *CYP1A2* (cytochrome P450 1A2) as a putative cause of the disorder, a potential target for clinical research and was consequently validated by immunofluorescence staining and electron microscopy.

In another study that relies on a rodent model system, Huang et al. explored the causes of acute temporal lobe epilepsy (a disorder that is difficult to diagnose) in a mouse epileptic model. They extracted data from the transcriptome and proteome of brain tissue to identify a set of initial causative candidates, which were further refined by machine learning methods to the three genes *Ctla2a*, *Hapln2*, and *Pecam1,* each with a remarkable predictability for the disorder.

The third selection in our Research Topic examined a set of innate and stress-induced anxiety-like behaviors in a mouse model (Gigliotta et al.). This study relied on the analysis of gene expression in the cortico-frontal and hippocampal regions of the brain. Compared with the non-anxious control, the anxious variant showed a pattern of gene expression enriched in inflammation and immunity processes. Moreover, they leveraged specialized databases and methods for the identification of drugs and compounds that are associated with the gene expression signatures, thereby offering a treatment direction for this disorder.

For the fourth article, Pan et al. analyzed A-to-I RNA editing in a mouse variant used in the study of myopia. Through RNA sequencing and its analysis, they identified a large set of these editing events, and their genes associated with “form-deprivation” myopia (retinal). These genes are also reported to vary in their roles across the stages of eye development. Their finding was supported through analysis of protein-protein interaction data and was consistent with previously reported literature.

Lastly, Audet et al. utilized whole genome, transcriptomic, and long-read sequencing for molecular diagnosis of late-onset ataxia. Among the eight patients who initially lacked a molecular-specific diagnosis despite clinical examination, several of them were subsequently diagnosed. Consequently, a set of novel genetic variants were identified, which demonstrates the translational potential of another multi-omics study with application to the clinical setting.

These studies of our Research Topic share the use of multi-omic techniques and reliance on bioinformatics for the analysis of large-scale data sets. The statistical power of “big data”, and *a posteriori* analysis by statistical methods coincides with recent innovations in the area of computer science known as deep learning. An example of its innovativeness is seen in Galactica (Taylor et al., 2022), a large language model, which is tailored to the natural sciences and has some capacity to “store, combine and reason about scientific knowledge”. The genetic-based studies of our Research Topic are adapted for this deep learning framework and its potential to automate the organization and analysis of large data sets (Fawzi et al., 2022).

Looking ahead, there is great promise in these multi-omic approaches and their large-scale datasets, where the data may serve as input to power modern machine learning architectures and systems. In particular, deep learning is continuing to enhance our capability at the genetic modeling of pathologies (Poplin et al., 2018; Jumper et al., 2021; Baid et al., 2023; Liao et al., 2023; Lin et al., 2023). As these models are often highly parameterized, they have been applied to the modeling of the complex interactions between candidate therapeutic agents and their molecular targets (Askr et al., 2023). While the underlying parameterization of these models is less tractable for validation, in itself an empirical science (Nanda et al., 2023), the deep learning approaches, when paired with “big data”, is powerful for the disentangling of the complexity often observed at the molecular and cellular level of biological systems.

Therefore, approaches that can pair multi-omic approaches with deep learning methods are suggested for the advancement of clinical research and a better understanding of the molecular mechanisms, along with the corresponding drug development, of diseases and disorders of the nervous system.
